# Supplementary material for: NPFF Decreases Activity of Human Arcuate NPY Neurons: A Study in Embryonic-Stem-Cell-Derived Model
Source: Int J Mol Sci. 2022 Mar 17;23(6):3260. doi: 10.3390/ijms23063260 (PMC8948797; doi:10.3390/ijms23063260)
Supplement: Supplementary file 1 [file ijms-23-03260-s001.zip › Supplemental Tables.pdf]

Supplemental Table S1. Comparison of percentages of mouse hypothalamic neurons expressing NPFF receptors in studies selected for the re-analysis (Data are shown as means ± SEM) [37–40].

	**Npffr1 ***	**Npffr2 ***	**Npffr1 + Npffr2 ***	**Adjust Factor ****	**Npffr2 Adjusted (Noffr2 x Adjusted Factor)**
Campbell et al., 2017	5.8	1.17	0.19	1.0	1.17
Kim et al., 2019	11.68	1.4	0.26	0.71	0.99
Mickelsen et al., 2019	8.28	0.0	0.0	0.64	0.0
Romanov et al., 2017	5.68	0.58	0.22	0.72	0.40

* % of neurons expressing gene or co-expressing genes. ** based on genes detected per cell.

Supplemental Table S2. Percentages of neurons expressing different neuronal markers in Npffr2-positive neuron population in mouse ARC (Data are shown as means ± SEM; data from re-analysis [37]).

**Gene**	**Number of Positive Cells**	**%**
Npffr2	152	100
Npy	98	64.47
Gad2	98	64.47
Gad1	97	63.81
Agrp	91	59.86
Pomc	61	40.13
Sst	43	28.28
Ddc	40	26.31
Slc17a6	33	21.71
Npffr1	25	16.45
Pnoc	24	15.79
Th	7	4.60

Supplemental Table S3. List of significantly enriched gene in *Npffr2*-positive compared to *Npffr2*-negative mouse arcuate neurons (Data are shown as means ± SEM) [37].

Gene	*p* Value	Mean log Fold Change	% of Npffr2+ Neurons Expressing Gene	% of Npffr2- Neurons Expressing Gene	*p* Value Adjusted
Npffr2	0	1.21	1	0	0
Acvr1c	1.94×10^{-35}	0.73	0.48	0.13	4.42×10^{-31}
Agrp	2.97×10^{-27}	1.03	0.60	0.24	6.77×10^{-23}
Serpina3n	6.00×10^{-25}	0.72	0.62	0.26	1.37×10^{-20}
Gm8773	7.90×10^{-22}	0.38	0.22	0.05	1.80×10^{-17}
Npy2r	2.88×10^{-20}	0.41	0.57	0.23	6.56×10^{-16}
Npy	1.09×10^{-18}	1.06	0.65	0.36	2.48×10^{-14}
Otp	6.38×10^{-18}	0.44	0.51	0.21	1.45×10^{-13}
Cited1	2.99×10^{-17}	0.58	0.80	0.51	6.82×10^{-13}
Csgalnact1	2.05×10^{-16}	0.26	0.31	0.10	4.67×10^{-12}
Hspb8	1.02×10^{-14}	0.27	0.28	0.09	2.32×10^{-10}
Irs4	1.22×10^{-14}	0.50	0.68	0.40	2.79×10^{-10}
Ghr	5.98×10^{-14}	0.36	0.41	0.17	1.36×10^{-9}
Vwa5a	7.39×10^{-14}	0.28	0.30	0.11	1.68×10^{-9}
Dgkk	9.15×10^{-14}	0.36	0.55	0.26	2.09×10^{-9}
Arhgef28	1.22×10^{-13}	0.40	0.42	0.18	2.78×10^{-9}
Hspa1a	1.86×10^{-13}	0.46	0.38	0.16	4.24×10^{-9}
Prune2	3.35×10^{-13}	0.39	0.66	0.35	7.63×10^{-9}
Gnal	2.43×10^{-12}	0.30	0.59	0.30	5.54×10^{-8}
Flrt3	6.49×10^{-12}	0.25	0.42	0.18	1.48×10^{-7}
Phf6	1.47×10^{-11}	0.35	0.39	0.17	3.36×10^{-7}
B2m	2.10×10^{-10}	0.27	0.54	0.29	4.78×10^{-6}
Rpl13	4.65×10^{-10}	0.30	0.74	0.46	1.06×10^{-5}
Angpt1	1.42×10^{-9}	0.27	0.22	0.08	3.23×10^{-5}
Ociad2	2.20×10^{-9}	0.34	0.64	0.39	5.01×10^{-5}
Arhgap6	1.36×10^{-8}	0.31	0.62	0.38	3.10×10^{-4}
Jun	1.91×10^{-8}	0.36	0.61	0.39	4.36×10^{-4}
Tmem255a	3.05×10^{-8}	0.28	0.73	0.46	6.96×10^{-4}
Dnm3	6.50×10^{-8}	0.29	0.81	0.58	1.48×10^{-3}
Arhgap36	6.63×10^{-8}	0.32	0.59	0.37	1.51×10^{-3}
1500012F01Rik	7.43×10^{-8}	0.33	0.72	0.50	1.69×10^{-3}
Gas5	8.82×10^{-8}	0.27	0.90	0.77	2.01×10^{-3}
Eef2	9.33×10^{-8}	0.27	0.88	0.66	2.13×10^{-3}
Rgs2	1.26×10^{-7}	0.43	0.73	0.55	2.88×10^{-3}
Ndn	2.49×10^{-7}	0.26	0.92	0.74	5.67×10^{-3}
Fos	8.08×10^{-7}	0.27	0.49	0.29	1.84×10^{-2}

Supplemental Table S4. Results from the main gene expression analysis. The Delta (ΔCT) relative quantification method was used to evaluate mRNA levels of target genes relative to a housekeeping gene (ACTB) in hNSC, hDHN and hALN (Data are shown as means ± SEM).

	hNSC (n=3)		hDHN (n=3)		hALN (n=2)	
**Gene ID**	**Mean**	**SEM**	**Mean**	**SEM**	**Mean**	**SEM**
AGRP	13.21	1.04	16.23	0.67	15.14	0.38
ASCL1	4.51	0.55	4.58	0.65	6.22	0.21
CARTPT	-	-	15.27~	~	11.30	0.30
CRH	7.66	0.19	12.96	1.03	12.80	0.20
DBX1	13.73	0.50	10.74*	0.09	13.98	0.15
EMX1	10.59	0.90	-	-	15.01~	~
EN2	15.42	0.78	15.89	0.72	14.01	0.23
FOXG1	4.13	0.52	4.75	0.10	6.83	0.01
GAD1	10.19	0.52	7.61	0.32	4.20	0.13
GAD2	10.70	0.23	7.26*	0.18	7.70	0.26
KISS1	-	-	-	-	15.65	0.63
MAP2	423	0.36	3.37	0.20	3.54	0.33
MCH	11.81	0.63	14.88	0.66	8.74	0.27
NEUN	10.21	0.57	10.83	0.27	9.13	0.24
NEUROD1	7.09	0.65	6.40	0.28	10.33	0.32
NEUROG3	9.83	1.61	12.65	1.57	15.08	1.12
NHLH2	9.86	1.69	12.32	0.92	12.11	0.10
NKX2.1	9.24*	0.52	7.17	0.05	8.46	0.88
NPFFR1	15.13	1.02	15.80	0.59	11.76	0.44
NPFFR2	12.88	0.91	13.67	0.24	9.99	0.67
NPY	11.58	0.28	14.38	0.31	9.65	0.39
OTP	15.71	1.25	13.90	0.32	7.75*	0.01
PAX6	3.45	0.45	7.25	0.20	7.23	0.01
POMC	12.24	1.66	-	-	13.29	0.50
POU5F1	10.01	2.38	12.33	1.77	11.47	0.11
RAX	13.94	1.16	15.56	0.35	15.43	0.06
SIM1	12.94	0.73	12.98	0.39	7.89	0.34
SLC17A7	13.14	0.77	13.50	0.24	12.39	0.04
SOX1	9.63	0.80	11.69	0.60	12.45	0.00
SST	9.46	0.29	8.38	0.27	8.55	0.21
TH	12.62	1.23	11.68 *	0.30	9.29*	0.11
TRH	11.08	1.10	11.39	0.26	6.94	1.12

- Undetermined Ct values after 40 cycles. ~ Only one Ct value available. * p value < 0.05.

Supplemental Table S5. List of primers used in this study.

Gene	TaqMan Reference
18S	Hs99999901_s1
ACTB	Hs01060665_g1
AGRP	Hs003614403_g1
ASCL1	Hs00269932_m1
CARTPT	Hs00182861_m1
CRH	Hs01921237_s1
DDX1	Hs01380082_m1
EMX1	Hs00417957_m1
EN2	Hs00171321_m1
FOXG1	Hs00702391_s1
GAD1	Hs01065893_m1
GAD2	Hs00609534_m1
KISS1	Hs00158486_m1
MAP2	Hs00258900_m1
MCH	Hs01041242_g1
NEUN	Hs02760627_g1
NEUROD1	Hs01922995_s1
NEUROG3	Hs01875204_s1
NHLH2	Hs00271585_s1
NKX2.1	Hs00968940_m1
NPFFR1	HS00223340_m1
NPFFR2	Hs01003259_m1
NPY	Hs00173470_m1
OTP	Hs00259528_m1
PAX6	Hs01088106_g1
POMC	Hs01596743_m1
POU5F1	Hs04995079_g1
RAX	Hs00429459_m1
SIM1	Hs00231914_m1
SLC17A7	Hs00220404_m1
SOX1	Hs01057642_s1
SST	Hs00356144_m1
TH	Hs00165941_m1
TRH	Hs00999489_m1
