# Supplementary figures and images for: NPFF Decreases Activity of Human Arcuate NPY Neurons: A Study in Embryonic-Stem-Cell-Derived Model
Source: Int J Mol Sci. 2022 Mar 17;23(6):3260. doi: 10.3390/ijms23063260 (PMC8948797; doi:10.3390/ijms23063260)

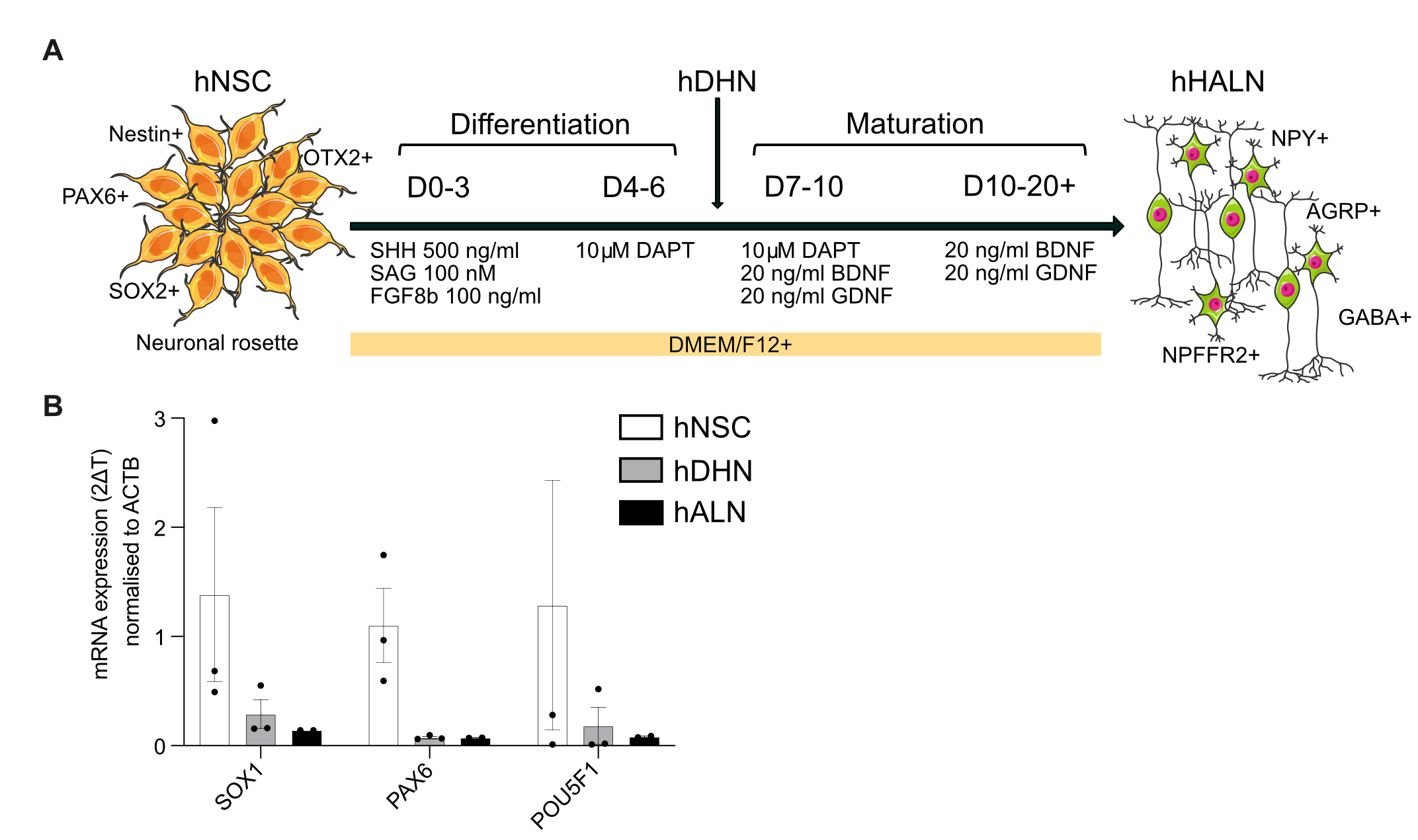

Supplement: Supplementary file 1 [file ijms-23-03260-s001.zip › Supplemental Figure S1.tiff]

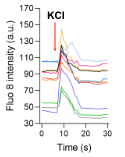

Supplement: Supplementary file 1 [file ijms-23-03260-s001.zip › Supplemental Figure S2.tiff]
